# Closing the gaps in patient management of dyslipidemia: stepping into cardiovascular precision diagnostics with apolipoprotein profiling

**DOI:** 10.1186/s12014-024-09465-w

**Published:** 2024-03-01

**Authors:** Esther Reijnders, Arnoud van der Laarse, L. Renee Ruhaak, Christa M. Cobbaert

**Affiliations:** Department of Clinical Chemistry and Laboratory Medicine, Leiden University Medical Center, Leiden, the Netherlands

**Keywords:** Apolipoproteins, Precision medicine, Apolipoprotein panel, Multiplex testing

## Abstract

In persons with dyslipidemia, a high residual risk of cardiovascular disease remains despite lipid lowering therapy. Current cardiovascular risk prediction mainly focuses on low-density lipoprotein cholesterol (LDL-c) levels, neglecting other contributing risk factors. Moreover, the efficacy of LDL-c lowering by statins resulting in reduced cardiovascular risk is only partially effective. Secondly, from a metrological viewpoint LDL-c falls short as a reliable measurand. Both direct and calculated LDL-c tests produce inaccurate test results at the low end under aggressive lipid lowering therapy. As LDL-c tests underperform both clinically and metrologically, there is an urging need for molecularly defined biomarkers. Over the years, apolipoproteins have emerged as promising biomarkers in the context of cardiovascular disease as they are the functional workhorses in lipid metabolism. Among these, apolipoprotein B (ApoB), present on all atherogenic lipoprotein particles, has demonstrated to clinically outperform LDL-c. Other apolipoproteins, such as Apo(a) - the characteristic apolipoprotein of the emerging risk factor lipoprotein(a) -, and ApoC-III - an inhibitor of triglyceride-rich lipoprotein clearance -, have attracted attention as well. To support personalized medicine, we need to move to molecularly defined risk markers, like the apolipoproteins. Molecularly defined diagnosis and molecularly targeted therapy require molecularly measured biomarkers. This review provides a summary of the scientific validity and (patho)physiological role of nine serum apolipoproteins, Apo(a), ApoB, ApoC-I, ApoC-II, ApoC-III, ApoE and its phenotypes, ApoA-I, ApoA-II, and ApoA-IV, in lipid metabolism, their association with cardiovascular disease, and their potential as cardiovascular risk markers when measured in a multiplex apolipoprotein panel.

## Introduction

Cardiovascular disease (CVD) is a leading cause of death worldwide and is associated with significant morbidity and mortality. In primary prevention the 10-year cardiovascular risk prediction model SCORE2 is applied which includes the following variables: non-high-density lipoprotein cholesterol (non-HDL-c), systolic blood pressure, sex, age, smoking status and geographical risk [1]. In secondary prevention patient management primarily relies on the conventional lipid panel, encompassing high-density lipoprotein cholesterol (HDL-c), triglycerides (TG), total cholesterol (TC), and calculated low-density lipoprotein cholesterol (LDL-c). If an individual is scored at high risk in primary or secondary prevention, the standard procedure is therapy with statins, which serve as the pharmaceutical cornerstone of cardiovascular therapy. Even if highly stringent LDL-c treatment target levels are met with statins, a substantial absolute risk of 70% of major adverse cardiovascular events (MACE) remains [2]. This “forgotten majority”, reflected by the substantial residual cardiovascular risk group beyond state-of-the-art treatment, has to be addressed!

### The tests don’t work

Diagnosis and patient management to suppress residual cardiovascular risk assessment should be improved. [3] Both direct and indirect tests of HDL-c and LDL-c are inherently flawed. As far back as 2010, Miller et al. assessed seven distinct direct tests to quantify HDL-c and LDL-c [4]. While most of these tests performed adequately when applied to normolipidemic samples from healthy individuals, they proved inadequate for individuals with abnormal lipid levels. This lack of specificity towards an atypical lipoprotein profile caused all seven tests to fail their analytical performance criteria. Under aggressive lipid lowering and in case of hypertriglyceridemia the measurement uncertainty of LDL-c tests at the low end is huge, making conventional LDL-c tests no longer fit for on-treatment monitoring of patients. Unfortunately, clinicians are not sufficiently aware about these test limitations and just treat the number. Instead of a meaningful test result that is measured accurately, based on a well-defined measurand. Therefore, a paradigm shift towards molecularly defined measurands and targeted therapies is required [5] as we need to understand what we are measuring to refine diagnoses and target therapies. Only then we contribute to precision diagnostics, taking into account the patient’s phenotype and interindividual variation.

Besides flawed tests for traditional lipids, emerging risk factors such as lipoprotein(a) (Lp(a)) have challenged analytical test development and performances. Lp(a) has emerged as a strong independent predictor of atherosclerotic cardiovascular disease (ASCVD) and aortic valve stenosis [6]. Despite the 2022 EAS consensus statement recommending for improved patient’s risk classification its measurement at least once in a lifetime, Lp(a) is not yet routinely measured in clinical practice [6]. The standardization of immunoassay-based Lp(a) tests is challenging because of the heterogeneity of the Apo(a) molecules both in patient specimens and in kit calibrators used [7–9].

### The drugs don’t work

Primary and secondary CVD prevention is focused on LDL-c lowering with statins as a first hit, which is further expanded with other therapeutic agents such as ezetimibe, proprotein convertase subtilisin/kexin type 9 (PCSK9) inhibitors alirocumab and evolocumab, or small interfering RNA (siRNA) inclisiran. These therapies only target one risk factor (LDL-c), while other lipid abnormalities, such as elevated Lp(a) levels, accumulation of remnant VLDL and/or chylomicron (CM) particles, increased levels of dysfunctional HDL, or familial dysbetalipoproteinemia, are neglected and therefore remain untreated.

Statins are widely prescribed as cardiovascular therapy, as recommended by large randomized controlled trials (RCTs) However, their clinical efficacy in terms of cardiovascular event prevention, side-effects, adherence, and toxicity are also questionable [10–12].

RCTs have a tendency to selectively include specific patient groups, introducing a selection bias that masks the true effectiveness of the novel therapeutic agent under study. Multiple examples of this phenomenon can be given. For example, Diamond and Ravnskov described that within the British Heart Protection Study, 26% of the participants allocated to simvastatin therapy were excluded during the run-in period, likely because of adverse events or lack of effects. Obviously, this introduces a serious bias [11]. If an RCT fails to demonstrate the efficacy of a therapeutic agent in a large group of subjects, subsequent post-hoc analyses on subgroups are performed to eventually find a selection of patients that may benefit from the novel therapy [11]. In addition, the results of many RCTs are reported as relative risk reduction in percentages between the study group and placebo group, which gives the impression of a substantial risk reduction. However, the absolute risk reduction may be marginal, typically around 1–2% for most cardiovascular RCTs [11]. Moreover, current treatment targets are based on the perfect average patient archetype; a Caucasian male between 40 and 60 years old and with only one health condition, while we are all aware that this average patient is not representative for the whole group of patients [12]. This approach is outdated and unjustifiable. As health care professionals, we must strive for a more tailored and personalized approach.

Over the years new therapies directly targeting molecularly defined biomarkers such as Apo(a), ApoC-III and ApoB have gradually made their way to the market or are currently in the process of being developed [13–15]. If the intention is to molecularly target these risk factors, then we must molecularly measure these factors as well. This involves medical tests that comply to both analytical performance and clinical performance criteria. We need to move towards molecularly defined health and disease criteria.

### Apolipoproteins are the future!

Apolipoproteins are key components of lipoprotein particles, which play a crucial role in lipid transport and metabolism [16]. Apolipoproteins are more and more recognized as important biomarkers for CVD risk assessment and disease management. In particular, apolipoprotein B (ApoB) has been identified as a strong independent predictor of CVD risk, with several studies demonstrating its superiority over traditional lipids such as LDL-c, and non-HDL-c [17–19]. Additionally, Apo(a), a characteristic apolipoprotein of Lp(a), attracted attention as well [6]. With the advent of precision medicine, there is a growing need for accurate and reliable biomarkers that support risk assessment and guide personalized treatment strategies. In this context, apolipoproteins have emerged as promising candidates for precision diagnostics, offering new opportunities for improved CVD risk management. We believe that the following apolipoproteins are most promising in cardiovascular risk management: Apo(a), ApoB, ApoC-I, ApoC-II, ApoC-III, ApoE, ApoA-I, ApoA-II, and ApoA-IV, including ApoE phenotype. Table 1 lists these apolipoproteins, the lipoproteins they reside on, whether they are exchangeable or not between these lipoproteins, and the major site of synthesis. Table 2 lists the receptors, enzymes and transporter proteins that interact with the apolipoproteins as part of the lipid metabolism. In this review we summarize the rationale for selecting the chosen nine apolipoproteins (apolipoprotein panel, or apo-panel) on lipid metabolism and their association with CVD. Awaiting the outcome of the apolipoprotein profiling performed in the ODYSSEY OUTCOMES trial regarding clinical effectiveness, relevant test indications will be deduced for this apolipoprotein panel as well as its test role in the clinical care pathway for cardiovascular precision medicine.

**Table 1:** Characteristics of apolipoproteins across the lipoprotein classes

Apo	Lipoproteins	Exchangeable?	Site of synthesis
ApoB100	VLDL, IDL, LDL, Lp(a)	No	Liver
ApoB48	CM, CM remnants	No	Intestine
Apo(a)	Lp(a)	No	Liver
ApoA-I	HDL, CM	Yes	Liver, intestine
ApoA-II	HDL, CM	Yes	Liver, intestine
ApoA-IV	HDL, CM	Yes	Intestine
ApoC-I	CM, VLDL, IDL, and HDL	Yes	Liver
ApoC-II	CM, VLDL, IDL, and HDL	Yes	Liver, intestine, macrophages
ApoC-III	CM, VLDL, IDL, and HDL	Yes	Liver
ApoE	CM, CM remnants, VLDL, IDL, HDL	Yes	Liver, intestine, macrophages, brain

**Table 2:** Receptors, enzymes and transporter proteins and their interaction either (+) activated or (-) inhibited by apolipoproteins in lipid metabolism

	Involved particle	Essential apo	Ref
**Receptor**			
LDLR	LDL	ApoB-100	[20]
	VLDL, IDL	ApoE ApoB-100 lesser degree than ApoE	[21]
	CM	ApoE	[21]
	Remnants	ApoE ApoB-100 lesser degree than ApoE	[21]
VLDLR	VLDL, IDL	ApoE	[21]
	CM	ApoE	[21]
	Remnants	ApoE	[21]
LRP1	VLDL, IDL	ApoE	[22]
	Remnants	ApoE	[21]
	CM	ApoE	[21]
SR-B1	HDL	ApoA-I Free ApoE	[23, 24]
**Enzyme**			
HL	VLDL, IDL	ApoE (+)	[25]
	HDL	ApoE (+) ApoC-I (-)	[26, 27]
LPL	VLDL, IDL	ApoC-I (-) ApoC-II (+ or - depending on its concentration) ApoC-III(-)	[28, 29, 30, 31, 32]
LCAT	HDL	ApoA-I (+) ApoA-IV (+) ApoC-I (+)	[27, 33]
**Transporter protein**			
ABCA1	HDL	ApoA-I (+) ApoE (+)	[34, 35]
CETP	VLDL, CM, HDL	ApoC-I on HDL (-)	[36]

## Biochemistry of apolipoproteins in lipid metabolism and their effect on cardiovascular disease

### ApoB

ApoB is an insoluble 550 kDa apolipoprotein, which cannot be exchanged between the particles of different lipoprotein classes. ApoB-100 is present on very-low density lipoprotein (VLDL), intermediate density lipoprotein (IDL), VLDL-remnants, LDL, and Lp(a), while its truncated form, ApoB-48, is only present on chylomicrons (CM) and their remnants.

#### Role of ApoB-100 in lipid metabolism

ApoB-100 is essential in the formation of VLDL particles. ApoB-100 is produced in the liver. Microsomal triglyceride transfer protein (MTP) enriches ApoB-100 with triglycerides (TG), resulting in pre-VLDL. Pre-VLDL is then further lipidated to its mature state, after which it is released into the circulation [20]. The primary function of VLDL is to transport TG to muscle cells and adipose tissue. The majority of VLDL is cleared from the circulation through lipolysis of TG by lipoprotein lipase (LPL), resulting in IDL which is further metabolized to either LDL or to VLDL remnants, which both contain an ApoB-100 molecule per particle [20]. A second metabolic pathway is direct clearance of VLDL or IDL via ApoE-mediated binding to the LDL receptor (LDLR), LDL-like receptor protein-1 (LRP1) and the VLDL receptor (VLDLR) [21]. LDL particles can only be cleared from the circulation via ApoB-100 recognition by the LDLR. VLDL remnant particles, which besides ApoB-100 also contain ApoE, can be cleared more easily through ApoE-mediated binding to several receptors: LDLR, LRP1 and VLDLR.

#### Role of ApoB-48 in lipid metabolism

ApoB-48 is essential in CM assembly, stability, and metabolism. After being synthesized, ApoB-48 is lipidated by MTP in intestinal cells to form nascent CM, followed by further processing, maturation, enrichment with ApoA-IV (see further), and entry into the circulation. Clearance of CM involves lipolysis of TG, similar to VLDL clearance, resulting in CM remnants. CM remnants are cleared by the liver through ApoE-mediated binding to LDLR, LRP1 and VLDLR [21]. Noteworthy, ApoB-48 itself is unable to interact with LDLR as it lacks the LDLR-binding domain present on ApoB-100 [20]. Accumulation of TGRLs and their remnants may cause atherosclerosis, as they are able to penetrate the arterial wall. In addition, they can cause inflammation and endothelial dysfunction contributing to cardiovascular risk [37–39].

#### ApoB-100 as cardiovascular risk marker

Recently, Mendelian randomization studies demonstrated that ApoB-100 is causally linked to the risk of coronary heart disease (CHD), coronary artery disease (CAD), acute coronary syndrome (ACS), and heart failure [40–42]. Genetic variants of ApoB also elucidated the role of ApoB in cardiovascular risk. For example, familial hypercholesterolemia (FH) is predominantly caused by a defective LDLR (90-95%). However, in a minority of cases (5–10%) FH is related to a defective ApoB-100 variant, which is associated with elevated levels of LDL-c and elevated risk of ASCVD. This is caused by a reduced binding affinity of mutant ApoB-100 to the LDLR, hampering the clearance of LDL particles from the circulation [43]. When comparing the elevated LDL-c levels observed in this ApoB-related FH to those in LDLR-related FH, the LDL-c levels are lower, which can lead to underdiagnosis of ApoB-related FH [43]. As to ASCVD risk prediction ApoB is highly correlated to the well-established biomarkers, LDL-c and non-HDL-c, it is recommended to implement ApoB in current clinical practice, since LDL-c and non-HDL-c are biologically and clinically less meaningful than ApoB [44]. Indeed, over the years several studies have reported that ApoB is more predictive than LDL-c and non-HDL-c [17–19]. For example, in the ODYSSEY OUTCOMES trial ApoB levels were associated with the risk of MACE, independent of LDL-c and non-HDL-c [17]. In addition, ApoB is molecularly defined and can be measured accurately and precisely unlike LDL-c and non-HDL-c [45]. This superiority is particularly evident at low LDL-c levels, where conventional LDL-c testing is insufficient, and at high TG levels that disturb calculation of LDL-c [5]. Overall, ApoB is a highly accurate marker for cardiovascular risk assessment, for treatment effect monitoring, and to examine whether targets are reached. An important advantage of the choice for ApoB is the fact that ApoB tests are robust and not matrix-sensitive, in contrast to LDL-c and non-HDL-c tests [5, 16, 45, 46].

### Apo(a)

#### Role of Apo(a) in lipid metabolism

Apo(a) is a plasminogen-like glycoprotein that is covalently bound to ApoB-100 in Lp(a), an LDL-like particle. Due to the presence of varying numbers of repeats of kringle-IV type 2 (KIV2)-encoding sequences, Apo(a) exhibits size polymorphism, which results in the existence of over 40 isoforms of Apo(a) [16]. Human kinetic studies have shown that Lp(a) assembly occurs both extracellularly as a product of LDL binding to Apo(a), as well as intracellularly where nascent Apo(a) and ApoB are assembled in the liver [47–49]. However, the exact mechanism of Lp(a) formation is not fully understood [20]. Neither is the clearance mechanism of Lp(a) fully understood. It has been proposed that Lp(a) is cleared from the circulation via the LDLR as Lp(a) resembles LDL [50]. However, conflicting results on the involvement of LDLR in Lp(a) clearance have been reported [50]. This is illustrated by the fact that both statins and PCSK9 inhibitors upregulate LDLR. If LDLR is involved in Lp(a) clearance, both therapies should reduce Lp(a) levels. Surprisingly, while PCSK9 inhibitors effectively lower Lp(a) levels, statins increase Lp(a) levels, suggesting that LDLR does not significantly contribute to Lp(a) clearance [51, 52]. Other receptors like scavenger receptor B1 (SR-B1), and the plasminogen receptor Plg-R_KT_ have been proposed as well [53]. However, it is still unclear whether these receptors are involved in Lp(a) clearance in humans.

The role of Lp(a) in normal physiology is still unknown. However, several mechanisms have been proposed for its role in pathophysiology. As Lp(a) resembles LDL, Lp(a) is thought to cause atherosclerosis by the same mechanism as LDL. In addition, the lysine-binding sites of Apo(a) have been shown to bind to damaged endothelium and to promote retention of Lp(a) in the arterial wall [54].

Furthermore, Lp(a) is the main carrier of proinflammatory oxidized phospholipids (OxPL). OxPL can be covalently bound to Apo(a) kringle-IV type 10. This OxPL-Apo(a) moiety has been shown to upregulate IL-8 expression [55]. Furthermore, OxPL-Apo(a) can induce multiple cellular cascades resulting in endothelial dysfunction, recruitment of monocytes, macrophage apoptosis, cytokine release, and smooth muscle cell migration and proliferation [53].

#### Apo(a) as cardiovascular risk marker

While the exact function of Lp(a) remains unclear, Lp(a) is an established causative risk factor for ASCVD and aortic valve stenosis [6]. Moreover, there is evidence suggesting an inverse correlation between Lp(a) levels and predisposition for type 2 diabetes [6]. Mendelian randomization studies have further demonstrated that elevated Lp(a) levels are causally linked to various conditions, including CHD, myocardial infarction, stroke, peripheral vascular disease, heart failure, and aortic valve stenosis [56, 57]. Randomized clinical trials with therapeutics directly targeting Lp(a), such as pelacarsen in Lp(a)HORIZON and olpasiran in OCEAN(a), are ongoing. While we await the full results from these trials, some insights have already emerged from randomized clinical outcome trials involving PCSK9 inhibitors. For example, the ODYSSEY OUTCOMES trial has demonstrated that lower Lp(a) levels are associated with a reduced incidence of MACE, independent of LDL-c [51, 58].

### ApoC-I

ApoC-I is a component of VLDL and HDL, primarily secreted by the liver [59–61]. ApoC-I is a highly exchangeable protein, enabling rapid dissociation from VLDL to associate with HDL, which is the main carrier of ApoC-I in normolipidemic plasma [61, 62]. The fraction of ApoC-I on non-HDL lipoproteins is minor (≈ 10–20%) compared with that on HDL (≈ 80–90%) [28, 63]. ApoC-I concentration is elevated in individuals with hypertriglyceridemia (HTG) and in patients with type III hyperlipoproteinemia, but not in persons with hypercholesterolemia. In the presence of elevated levels of TG, for example in type 2 diabetes mellitus (T2DM) patients, the distribution of ApoC-I is shifted from HDL towards TGRLs [64]. The distribution of ApoC-I on different particles is important, as ApoC-I exhibits an ambiguous role in lipid metabolism depending on the particle it resides on.

#### ApoC-I in HDL metabolism

When residing on HDL, ApoC-I increases HDL-c by the following properties: (1) inhibition of cholesteryl ester transfer protein (CETP) [36], (2) inhibition of hepatic lipase (HL) [26], (3) activation of lecithin–cholesterol acyltransferase (LCAT) [65, 66], and (4) reduction of SR-B1–mediated uptake of HDL-c [67]. Inhibition of CETP is proposed to occur because of a change in electrostatic charge of the HDL particle inflicted by ApoC-I, resulting in a weaker interaction between HDL and CETP, and hindering cholesterol ester transfer from HDL to other lipoproteins [68]. Besides CETP inhibition, ApoC-I is able to inhibit HL, which converts larger HDL_2_ particles into smaller HDL_3_ particles [69, 70]. Furthermore, ApoC-I is able to activate LCAT in vitro, initiating cholesterol esterification, resulting in mature HDL [65, 66]. By activating LCAT and inhibiting CETP and HL, ApoC-I facilitates the synthesis and stabilization of mature HDL particles, resulting in elevated levels of HDL-c [36, 68, 71]. Finally, high levels of ApoC-I have shown to reduce SR-B1–mediated uptake of cholesterol esters from HDL [67]. All these processes result in an increase of HDL-c, suggesting a cardioprotective role for ApoC-I on HDL.

#### ApoC-I in TGRL metabolism

In case ApoC-I resides on TGRLs, ApoC-I has a completely different role than when residing on HDL. ApoC-I on TGRL (1) loses its ability to inhibit CETP, leading to lower plasma HDL-c levels [72], (2) inhibits LPL activity, delaying TGRL hydrolysis [28, 29], (3) displaces ApoE from TGRLs, inhibiting ApoE-mediated binding and clearance of TGRLs and IDLs by VLDLR [73], LDLR [74], and LRP1 [75], and (4) increases VLDL-TG and VLDL-ApoB production [61]. Interestingly, in patients with elevated TG (e.g. DM patients [76] and CAD patients with HTG or CAD patients with combined hyperlipidemia [77]) CETP activity is positively correlated with ApoC-I concentrations, whereas in healthy controls this association was absent. The distribution of ApoC-I in DM patients, favoring TGRLs over HDL, provides a plausible explanation for the positive correlation between ApoC-I concentrations and CETP activity observed in patients with DM. Furthermore, ApoC-I on TGRLs is able to inhibit LPL activity by displacing LPL from these particles, thereby impairing TG hydrolysis, resulting in delayed clearance of TGRLs [28, 29]. This inhibition was shown to be independent of ApoC-III in transgenic mice models, another inhibitor of LPL [78]. In addition, ApoC-I is able to displace ApoE from TGRLs and/or change the conformation of ApoE [71], inhibiting ApoE-mediated binding and clearance of TGRLs and remnants by VLDLR [73], LDLR [74], and LRP1 [75]. Finally, in ApoE deficient mice it was demonstrated that ApoC-I was able to increase the VLDL-TG and VLDL-ApoB production [28], and this has also been observed in HTG patients [61]. To summarize, ApoC-I is able to inhibit LPL, HL, and subsequent clearance of TGRLS.

#### ApoC-I as cardiovascular risk marker

ApoC-I has a dual role in lipoprotein metabolism and cardiometabolic risk. When residing on TGRLs, ApoC-I delays the plasma clearance of TGRLs and, as such, ApoC-I is harmful and promotes cardiometabolic risk; however, when residing on HDL, ApoC-I increases plasma HDL-c and is considered protective [36, 67, 69, 79].

In patients with CHD and hyperlipidemia the inhibition of plasma CETP by ApoC-I is blunted, which is probably due to increasing amounts of VLDL-bound ApoC-I which is less active as inhibitor of CETP than HDL-bound ApoC-I [77]. The HDL of patients with CHD contains less ApoC-I (by down-regulation) than the HDL of healthy controls [80]. While TGRLs are normally taken up by ApoE-mediated binding to liver receptors, this process is inhibited by ApoC-I and ApoC-III [81]. Enrichment of TGRLs with ApoC-I is associated with a proatherogenic composition of the particles due to increased cholesterol/TG ratio related to prolonged half-life of TGRL remnants in the circulation [82]. Postprandial TGRL is enriched with ApoC-I in patients with CAD, and in healthy individuals with increased intima media thickness (IMT) [83]. In normolipidemic healthy middle-aged men postprandial TGRL enriched with ApoC-I is an independent predictor for IMT [82]. These findings suggest that the ApoC-I content of TGRL is a risk factor for early atherosclerosis and CAD [84]. In patients with carotid atherosclerosis the total plaque area increased linearly with the number of ApoC-I molecules per VLDL-particle both in the fasting and the postprandial state. Thus, there is ample evidence for a pivotal role for the number of ApoC-I molecules per VLDL-particle in initiation and progression of atherosclerosis [84].

Quantitative proteomics revealed that ApoC-I, ApoC-II and ApoE were elevated in patients with myocardial infarction [85]. Furthermore, in the PROCARDIS study involving patients with CHD, ApoC-I, ApoC-III and ApoE were found to be associated with CHD, as measured by quantitative proteomics [86].

### ApoC-II

ApoC-II is mainly produced in the liver and intestine and assembles with VLDL, IDL, CM and HDL particles [87].

#### Role ApoC-II in lipid metabolism

Acting as an essential cofactor to activate LPL, ApoC-II is necessary for the hydrolysis of TG into free fatty acids (FFA) in TGRLs. After lipolysis, ApoC-II dissociates from the TGRL and moves to HDL, which serves as a storage site for ApoC-II until new TGRLs enter the circulation. Once new TGRLs appear, ApoC-II transfers from HDL to these particles to initiate the process once again [88]. The exact mechanism by which ApoC-II activates LPL is unknown. It has been proposed that ApoC-II supports LPL, as it binds TGRL and facilitates the entry of TGs into the active site of LPL, enabling efficient TG hydrolysis [88, 89]. In addition, Kumari et al. showed that ApoC-II was able to stabilize LPL and protect it from unfolding. Moreover, ApoC-II provides stability to sites involved in the sites anchoring the protein lid, whereas the LPL inhibitor ANGPTL3 was found to destabilize these same regions [90]. This might suggest why ApoC-II acts as an activator, and ANGPTL3 as an inhibitor of LPL.

#### ApoC-II as cardiovascular risk marker

ApoC-II deficiency can cause impaired clearance of TGRLs, leading to the accumulation of TGRLs, resulting in severe HTG. Interestingly, as early as 1994, it was reported that transgenic mice overexpressing human *APOC2* also exhibited HTG [30]. Apparently, there is an optimal ApoC-II concentration, which has been observed in clinical studies as well. Epidemiologic studies showed that low ApoC-II levels in intermediate-to-high risk patients were associated with risk of cardiovascular mortality [31]. Interestingly, the association between ApoC-II levels and the risk of cardiovascular mortality followed an inverse J-shaped curve, with the highest risk at the lower quintile (≤ 28.3 mg/L) and moderate-to-high risk in the upper two quintiles of ApoC-II levels (≥ 46.2 mg/L), whereas in the middle quintiles the risk was low [31]. These findings indicate the presence of an optimal ApoC-II level, highlighting that high ApoC-II levels do not necessarily imply better outcomes. Hermans et al. observed in the MISSION! Intervention Study that in 38 patients with premature CAD, 11% were found to have low ApoC-II levels (≤ 5.0 mg/L) with normal TG levels [91]. Despite their low a priori risk score for CAD, these patients presented with ST-segment elevation myocardial infarction and had a high relative risk of 10-year reinfarction or revascularization [91]. This particular phenotype of relatively young female patients with CAD has not been recognized earlier and deserves further study. Conversely, LPL activation was impaired at high concentrations of ApoC-II. Thus, it appears that ApoC-II does not function as a true activator of LPL, as elevated concentrations actually impair its activity. This finding aligns with the observations of Shachter et al. in transgenic mice overexpressing human *APOC2* [30]. They showed that in these mice VLDL particles were enriched in ApoC-II and depleted in ApoE. These VLDL particles with increased ApoC-II/ApoE ratio poorly bind to heparin, and this effect might also extend to the interaction of VLDL with lipases or receptors at the cell surface, impairing the clearance of TGRLs, ultimately leading to HTG [30]. ApoC-II as a therapeutic target might prove to be difficult due to the potential risk of overshooting the desired level, i.e. the optimal ApoC-II concentration.

### ApoC-III

ApoC-III is primarily associated with CM, VLDL, IDL, remnants, HDL and to a lesser extent with LDL particles [92]. In normal conditions ApoC-III is mainly associated with HDL, while in HTG patients it is mainly associated with TGRLs [92].

#### Role of ApoC-III in TGRL metabolism

The mechanisms by which ApoC-III influences TGRL metabolism are not fully understood. However, there is consensus that ApoC-III acts as an inhibitor in both the LPL-dependent and LPL-independent metabolic pathways.

The inhibition of LPL-mediated lipolysis of TG from TGRLs by ApoC-III was examined in human kinetic studies. These studies showed that loss of function (LOF) *APOC3* resulted in better clearance of VLDL-TG, compared to individuals with normally expressed *APOC3*. The direct clearance of VLDL particles was not affected, indicating an inhibitory role of ApoC-III on LPL [32]. It is suggested that ApoC-III prevents LPL from binding to TGRLs, after which ANGPTL4 inactivates LPL [93].

The role of ApoC-III in LPL-independent pathways is demonstrated in familial chylomicronemia syndrome (FCS) patients lacking LPL or LPL activity. In these patients, ApoC-III impairs the ApoE-mediated hepatic uptake of TGRLs. Administration of volanesorsen, an ASO directed at ApoC-III, led to reduction of TG levels in these individuals [94]. This suggests a role for ApoC-III in the LPL-independent clearance of TGRLs by the liver [32, 95]. In individuals with normal TG levels, the clearance of TGRLs occurs mainly through the binding of ApoE to hepatic receptors. However, in HTG patients with TGRLs enriched with ApoC-III, the clearance is primarily affected by ApoC-III, leading to a reduced clearance rate [93, 96]. This may be caused by ApoC-III’s ability to displace ApoE on TGRLs, preventing ApoE-mediated binding to the hepatic receptors.

The role of ApoC-III in the assembly and production of VLDL is controversial. Several studies in mice overexpressing human *APOC3* have shown an increase in VLDL production [92]. However, when examined in human studies involving individuals with either complete or partial LOF *APOC3*, normal rates of VLDL secretion were observed, suggesting that ApoC-III plays no significant role in VLDL assembly and secretion [32]. However, in overweight men hepatic secretion of VLDL was increased by ApoC-III [97].

#### Role of ApoC-III in HDL metabolism

In addition to its role in TGRL metabolism, ApoC-III may affect HDL metabolism. The number of ApoC-III molecules per HDL particle may vary. ApoC-III interacts with ApoE on HDL, mitigating the beneficial features of ApoE regarding cholesterol efflux [98, 99]. In addition, human ApoC-III can bind murine SR-B1 receptors [23]. ApoC-III-enriched HDL is associated with an increased risk of CHD compared to ApoC-III-free HDL [100]. Moreover, ApoC-III-enriched HDL was associated with Alzheimer’s Disease [95, 101].

In addition to its role in TGRL and HDL metabolism, ApoC-III plays a role in several other atherogenic processes by promoting monocyte adhesion, endothelial dysfunction, and pro-inflammatory processes [92]. ApoC-III has also been reported to facilitate LDL retention in the arterial wall [20, 102].

#### ApoC-III as cardiovascular risk marker

LOF mutations of *APOC3* are associated with lower plasma levels of TG, remnant cholesterol, total cholesterol and ApoC-III levels, compared to healthy individuals without mutated *APOC3* [95, 103–106]. Interestingly, carriers with LOF *APOC3* exhibit higher levels of HDL-c [106] and have a 40% lower risk of ASCVD compared to non-carriers [107, 108].

In the PROCARDIS case-control study for risk prediction of CHD, Clarke et al. investigated the relevance to determine the levels of thirteen individual apolipoproteins [86]. A strong positive association between ApoC-III and the risk of CHD was observed, independent of TG levels and other lipid parameters [109, 110]. In addition, van Capelleveen et al. and Katzmann et al. showed in CAD patients that ApoC-III was an independent predictor of cardiovascular events [109, 111]. Recently, therapeutics targeting ApoC-III have come to the market. In patients with FCS, volanesorsen has been reported to reduce ApoC-III levels by 90% [94]. However, the Food and Drug Administration (FDA) did not approve volanesorsen due to adverse events observed in the APPROACH trial. The European Medicine Agency (EMA) on the contrary did approve volanesorsen in FCS patients only. A GalNAc conjugated form of volanesorsen, olezarsen, showed a 74% decrease in ApoC-III levels in individuals with moderate HTG at high cardiovascular risk or with prevalent CVD [13]. Whether ApoC-III lowering improves clinical outcome is yet unknown.

### ApoE

ApoE is associated with VLDL, IDL, HDL, CM, and CM remnants. Plasma ApoE is synthesized primarily by liver hepatocytes, which account for ∼75% of the ApoE production. The second most common organ synthesizing ApoE is the brain. Here, ApoE is synthesized in situ and does not cross the blood brain barrier.

#### Role of ApoE in TGRL metabolism

ApoE plays an important role in TGRLs clearance, since it facilitates the binding to LDLR, LRP1, heparan sulfate proteoglycans (HSPGs), and VLDLR and therefore promoting their clearance. Clearance of TGRLs is relatively fast as compared to LDL, which is attributed to the presence of ApoE in TGRLs. ApoE can interact with LDLR with a higher binding affinity than ApoB-100, and thus is capable of regulating the levels of the lipoproteins on which it resides (VLDL and their remnants and CM remnants) as well as the levels of lipoproteins on which it does not reside (LDL) [112]. ApoE-mediated binding to LRP1 in the HSPG/LRP1 pathway initiates remnant lipoprotein clearance in the liver.

#### Role of ApoE in HDL metabolism

In addition to TGRLs, ApoE also resides on HDL where it plays a role in reverse cholesterol transport. ApoE binds the ATP-binding cassette transporter A1 (ABCA1) regulating the cholesterol influx and efflux of HDL [113]. ABCA1 binding is not affected by ApoE isoforms, hence all isoforms are equally effective in ABCA1-mediated cholesterol efflux [114].

#### ApoE and macrophages

ApoE is also expressed in macrophages, promoting cholesterol efflux via this way as well. Cholesterol efflux from macrophages is dependent on ApoE isoforms, of which ApoE2 and ApoE4 are associated with lower efflux compared to ApoE3 [115]. This can result in the accumulation of cholesterol, foam cell formation and eventually inflammasome activation, all contributing to an increased ASCVD risk [115]. In addition, ApoE is able to reduce macrophage-mediated LDL oxidation of which the effectiveness seems to be dependent on ApoE isoforms, although conflicting results have been reported [115].

#### ApoE genotypes/phenotypes

ApoE is a polymorphic protein arising from three alleles: ε2, ε3 and ε4, which occur at different frequencies in humans and varies slightly among ethnic groups (ε2, 8–10% ; ε3, 70%; and ε4, 15–20% in Caucasians) and give rise to three homozygous (ApoE2/2, ApoE3/3, and ApoE4/4) and three heterozygous (ApoE3/2, ApoE4/2, and ApoE4/3) phenotypes [112, 116]. ApoE3 seems to be the normal isoform in all known functions, while ApoE4 and ApoE2 can each be dysfunctional. ApoE3 and ApoE4 bind to LDLR with similar affinity (∼20-fold greater than that of ApoB-100, the other LDLR ligand) [20]. ApoE2, however, defectively binds to the LDLR (∼2% of normal activity), because it has a cysteine at residue 158 instead of an arginine as in ApoE3 and ApoE4. ApoE4 increases plasma LDL levels and the risk for ASCVD [115]. ApoE2 and ApoE3 preferentially bind to HDL, whereas ApoE4 prefers to bind to VLDL and CM remnants [115]. The enrichment of VLDL with ApoE4 accelerates their clearance from plasma by receptor-mediated endocytosis in the liver; as a result, LDLR is downregulated, and plasma LDL levels increase.

#### ApoE as cardiovascular risk marker

It is generally considered that ApoE protects against the development of atherosclerosis, but this benefit depends on the ApoE isoform, the total plasma ApoE level, and the cell type responsible for the synthesis and secretion of ApoE. It is clear that the absence of ApoE is associated with increased risk; however, having too much ApoE may also be associated with increased risk. The role of high levels of ApoE in inhibiting lipolysis or increasing VLDL production may indicate an increased ASCVD risk as those TGRLs could contribute to the formation of atherogenic remnant particles. It is quite likely that there is an optimal range of ApoE plasma levels, and that levels above or below that range impose a risk rather than a benefit for atherosclerosis [117].

Generally, individuals with ε2 genotype have lower levels of LDL, but higher plasma levels of other lipoproteins and TG [115]. Because ApoE2 binds defectively to LDLR, ApoE2 homozygosity may precipitate type III hyperlipidemia. This disorder occurs only when another condition -diabetes, obesity, hypothyroidism, or estrogen deficiency- leads to overproduction of VLDL or fewer LDLR, overwhelming the limited ability of ApoE2 to mediate the clearance of TGRLs, thereby increasing the risk for atherosclerosis [118]. Nearly all patients with type III hyperlipidemia are homozygous for ApoE2. However, not all ApoE2 homozygotes have type III hyperlipidemia. In fact, most E2/E2 subjects (> 90%) are normolipidemic or even hypolipidemic, owing to reductions in LDL or HDL or both. The defective binding of ApoE2 to LDLR results in a delayed clearance of TGRLs, however this is usually insufficient to precipitate the disorder. A reason for this phenomenon may be the presence of a second lipoprotein receptor system involving HSPG/LRP, with which ApoE2 functions more efficiently than with the LDL receptor.

ApoE4 carriers have the highest risk to develop heart disease. In normolipidemic individuals, ApoE4 is associated with increased levels of TC, LDL-c and ApoB, whereas ApoE2 was associated with a reduced risk. In addition, clinical studies have shown that ApoE4 is overrepresented in both hyperlipidemic and heart disease populations [119–122]. For example, large vessel disease, myocardial infarction and stroke risks were shown to be higher in ε4 allele carriers than ε2 allele carriers [123, 124]. Several studies estimated a 40% higher risk for CHD mortality in ε4 carriers compared with ε2 carriers or carriers of the ε3/ε3 genotype [125]. These facts sustain the nowadays increased need for personalized medicine and treatment, based not only on marker levels in plasma, but also on genetic characteristics of each individual.

##### ApoE and neurological diseases

Apart from its role in ASCVD, ApoE also exhibits significant effects on neurological diseases. For example, carriers of the apo ε4 allele are associated with an increased risk of Alzheimer’s disease, frontotemporal dementia, Down syndrome, certain patients with Parkinson’s disease, and Lewy body disease [115, 126–129].

##### ApoE and lp(a)

*APOE* genotypes also have its effect on Lp(a) and ApoB levels. ApoE2/E2 was shown to be associated with the lowest levels of Lp(a) and ApoB, whereas ApoE4/E4 showed the highest levels of Lp(a) and ApoB [130]. This may be explained by Lp(a) competing for the same receptors as ApoE. ApoE2 is known to have defective binding and a low binding affinity for LDLR, which may enhance the clearance rate of Lp(a). This could be further exemplified by the fact that ApoE2 is associated with lower levels of LDL, which means less competition for Lp(a) in case it shares the same receptors as LDL [130]. On the other hand, ApoE4 has a high affinity for LDLR and LRP1, and is associated with increased levels of LDL. This may lead to outcompeting Lp(a) binding to the same receptors, ultimately resulting in elevated levels of Lp(a).

### ApoA-I

ApoA-I, synthesized mainly in the liver and small intestine, serves as the main structural component of HDL and represents 70% of the total protein content of HDL [131]. One HDL particle contains 2–5 ApoA-I molecules, depending on the size of HDL [132]. ApoA-I exhibits two distinct conformations: lipid-bound and lipid-free. The lipid-free form of ApoA-I accounts for approximately 8% of its overall concentration [133, 134].

#### Role of ApoA-I in lipid metabolism

ApoA-I plays a key role in the reverse cholesterol transport by which excess cholesterol is removed from peripheral tissues and transported back to the liver for excretion [135]. Lipid-free ApoA-I interacts with ABCA1, which is located in foam cells, liver, intestine, placenta, brain, and kidneys [20]. After interaction, ApoA-I facilitates the transfer of free cholesterol and phospholipids, resulting in the formation of nascent HDL [136]. Lipid-bound ApoA-I can then interact with LCAT, initiating cholesterol esterification, resulting in mature HDL. Mature HDL particles can now bind to ABCG1, ABCG4 and SR-B1, taking up cholesterol from foam cells residing in the arterial wall, hence stabilizing vulnerable plaques [20, 131]. Subsequently, cholesterol-rich HDL can either interact directly with hepatic SR-B1 to unload cholesterol esters, phospholipids and TG to complete reverse cholesterol transport, or transfer cholesterol esters to VLDL, IDL and LDL by CETP [131, 132]. Delipidated HDL can re-enter the cycle or be excreted by the kidneys.

#### ApoA-I as cardiovascular risk marker

Mendelian randomization studies identified an inverse relationship between ApoA-I and risk of CHD [40]. In addition, ApoA-I mutations have been linked to low levels of HDL and dysfunctional HDL, inflammation, defective LCAT activation, amyloidosis and overall increased risk of ASCVD [131, 137]. However, cardioprotective mutations have been reported as well. ApoA-I Milano is a genetic variant of ApoA-I, resulting in decreased levels of ApoA-I and HDL. Surprisingly, this genetic variant is also associated with a decreased risk of ASCVD [138].

Low levels of HDL and ApoA-I are associated with an increased risk of ASCVD. It was therefore unexpected that raising HDL levels, and consequently raising ApoA-I by CETP inhibitors in clinical trials, did not result in lower risk of CVD [139]. More recently, a shift in thinking has emerged, suggesting that the focus should not solely be on increasing HDL levels, but rather on increasing the functional subspecies of HDL. In 2022, Furtado et al. reported that CETP inhibitors did indeed increase ApoA-I levels, but it mostly increased ApoA-I in dysfunctional HDL subspecies that are associated with an increased risk of CHD [140]. This could explain why CETP inhibitors did not improve cardiovascular outcome. These dysfunctional HDL subspecies include HDL particles that contain ApoC-III in the presence and absence of ApoE. ApoC-III might displace ApoE on HDL particles, similarly as described for TGRLs, impairing the ApoE-mediated binding of HDL to liver receptors [140].

### ApoA-II

ApoA-II is the second most abundant protein on HDL accounting for 20% of the total protein content [141]. HDL can be categorized in HDL particles with ApoA-I (LpA-I), containing an average of three to four ApoA-I molecules, or a combination of ApoA-I and ApoA-II (LpA-I/A-II), containing two ApoA-I molecules and one dimer ApoA-II molecule (with a fixed molar ratio of 2:1) [142, 143]. ApoA-II presents itself in different quaternary structures, including monomers, homodimers, and heterodimers with ApoE and ApoD [144]. ApoA-II is primarily synthesized in the liver, and a minor fraction in the intestines [144].

#### Role of ApoA-II in lipid metabolism

ApoA-II dimerizes after lipid loading and is released as LpA-II in circulation. Subsequently, LCAT combines LpA-II with circulating LpA-I particles to form LpA-I/A-II particles [144]. Unlike ApoA-I, ApoA-II cannot activate LCAT [145, 146]. ApoA-II plays a role in HDL maturation and reverse cholesterol efflux and exerts antioxidative properties. The majority of ApoA-II molecules in HDL are found in association with ApoA-I, with only a small fraction of HDL that contains ApoA-II only (LpA-II). The concentration of LpA-I/A-II is constant regardless of HDL concentration, suggesting that increases in HDL levels is attributed to an increase in LpA-I, but not LpA-I/A-II [142]. Melchior et al. reported that LpA-I and LpA-I/A-II particles exhibit different proteomes [147]. They showed that the presence of ApoA-II attracts proteins mostly associated with lipid transport, whereas the absence of ApoA-II in LpA-I results in a proteome that favors inflammatory pathways, hemostasis, immune response, metal ion binding and protease inhibition [147]. For example, they observed that ∼90% of LCAT and CETP was associated with LpA-I/A-II and ∼10% with LpA-I. In addition, LpA-I/A-II increased the ABCA1-mediated cholesterol efflux from macrophages to the LpA-I/A-II particle, independently of other proteins on the particle [147]. This suggests that ApoA-II might be able to displace ApoA-I from HDL affecting the ApoA-I-mediated binding to ABCA1. Interestingly, plasma levels of LpA-I/A-II are positively associated with ApoB-containing particles, whereas there is an inverse relationship for LpA-I and ApoB, suggesting that LpA-I/A-II could be a marker for increased cardiovascular risk, while LpA-I is an antiatherogenic marker [143].

#### ApoA-II as cardiovascular risk marker

In contrast to ApoA-I, the role of ApoA-II in ASCVD has been poorly understood despite intensive studies. This is partly because of the structural differences between human ApoA-II and murine ApoA-II, which makes it difficult to extrapolate results obtained from mice to human [144, 148]. Conflicting results have been reported in terms of ApoA-II and the risk of ASCVD. For instance, individuals carrying the *APOA2* variant rs5082 (265T/C), which leads to lower ApoA-II levels, have shown a decreased risk of CAD [144, 149]. Conversely, complete ApoA-II deficiency does not appear to affect CAD risk at all [150, 151]. It is important to note that ApoA-II deficiency is rare, which requires caution when drawing conclusions based on the limited number of cases available.

Contrary to the previously mentioned findings, a large body of evidence suggests that elevated levels of ApoA-II are associated with a decreased risk of CAD, despite an increased risk of HTG [152–154]. For example, in the Prospective Epidemiological Study of Myocardial Infarction (PRIME) trial both LpA-I and LpA-I/A-II concentrations were inversely associated with the risk of CHD [155].

### ApoA-IV

ApoA-IV is associated with CM and HDL, or circulates in its unbound lipid-free form [156]. ApoA-IV is produced in the small intestine enterocytes and is secreted into intestinal lymph during fat absorption [157]. The findings regarding the distribution of ApoA-IV across various lipid particles are inconsistent. Some studies suggest that a significant portion of ApoA-IV exists in a lipid-free state, while others attribute the majority of ApoA-IV to HDL or CMs [156, 158–163]. Lipid-free ApoA-IV is primarily present as homodimer [157].

#### Role of ApoA-IV in chylomicron metabolism

ApoA-IV assembles with nascent CMs, which are eventually drained into the circulation through the thoracic duct. The TGs present in CMs undergo hydrolysis by LPL, after which most of ApoA-IV dissociates from the particle. This dissociated ApoA-IV either remains as lipid-free ApoA-IV or transfers to HDL. The exact reason why ApoA-IV dissociates from chylomicron remnants is not fully understood, but it is speculated that it may be due to competition with other apolipoproteins, namely ApoE and ApoC’s, which are also found on the surface of the remnants [157, 164].

#### Role of ApoA-IV in HDL metabolism

ApoA-IV is evenly distributed among LpA-I and LpA-I/A-II particles [147]. In vitro experiments have demonstrated that ApoA-IV can activate LCAT thereby promoting cholesterol esterification [165]. ApoA-I and ApoA-IV are the two most efficient co-factors for LCAT activity [166], however, the acyl donor specificity of ApoA-IV differs from that of ApoA-I [165]. Furthermore, in ApoA-I deficient individuals, it has been shown that HDL with ApoA-IV is able to take up and esterify cell-derived cholesterol, suggesting an important role for ApoA-IV in reverse cholesterol transport [162]. In addition, HDL-sized lipoprotein particles from ApoA-IV transgenic mice conferred greater ability to reduce cholesterol levels than those from wild type mice, possibly by increased esterification due to LCAT activation [167].

Besides LCAT activation, human ApoA-IV overexpression in transgenic mice was able to promote cAMP-sensitive cholesterol efflux from macrophages [164]. Analysis using N-terminal or C-terminal deletion mutants of ApoA-IV revealed that the C-terminal domain (aa 333–376) inhibits ApoA-IV’s ability to promote cholesterol efflux [168].

#### ApoA-IV as cardiovascular risk marker

ApoA-IV is generally considered an atheroprotective factor. In a genome-wide association meta-analysis focused on ApoA-IV concentrations, two genetic regions within the *APOA5-A4-C3-A1* cluster and one in *KLKB1* were identified to be associated with ApoA-IV concentrations [169]. Moreover, it was identified that 30% of the variation in ApoA-IV concentration is genetically regulated, and genetic variants could be associated with kidney function, HDL-c and TG levels [170]. Seven genetic variants of ApoA-IV have been identified in humans: ApoA-IV-1, ApoA-IV-1 A, ApoA-IV-2, ApoA-IV-2 A, ApoA-IV-3, ApoA-IV-0, and ApoA-IV-5, of which ApoA-IV-1 is the most common variant [171]. In comparison to ApoA-IV-1, ApoA-IV-1 A is associated with lower plasma ApoA-IV levels and higher risk of CAD [172]. Carriers of ApoA-IV-2 A showed increased HDL-c, and reduced LDL-c, TG and ApoA-I levels [171].

Several observational studies have consistently shown that low levels of ApoA-IV are associated with the risk of ASCVD, independent of classical lipids including HDL [86, 163, 173].

The distribution of ApoA-IV on the different lipid fractions is the same for CAD patients as for healthy controls [156]. This suggests that the lower ApoA-IV concentration has no effect on the distribution of ApoA-IV and that this distribution does not seems to affect the ASCVD risk in CAD patients.

## Potential added value of multiplex apolipoprotein testing

### Why measure apolipoproteins at all?

In this era of precision medicine the safe and effective management of dyslipidemia in all individual patients requires a more refined approach than what can be accomplished with the classical lipid panel of LDL-c, HDL-c, TG and TC. With expanding knowledge, a paradigm shift from the conventional lipid panel to a more refined approach with biologically and clinically more meaningful biomarkers is required to enable better cardiovascular risk stratification in the context of precision medicine. Apolipoproteins are the functional proteins in the lipid metabolism as outlined in the body of the review, and will likely serve as better indicators of lipoprotein functionality, and thus more effective predictors of cardiovascular disease [18, 44, 45, 174]. ApoB has already demonstrated its superior predictive value in comparison with LDL-C. Moreover, protein measurands can be much more unequivocally defined, resulting in tests with improved analytical specificity and analytical performance. Because of this, the tests that measure apolipoproteins are expected to outperform the classical tests with the conventional lipid panel. In addition, drugs targeting individual apolipoproteins are emerging in the market. Examples are olezarsen, an antisense oligonucleotide directed at ApoC-III, and olpasiran, a siRNA directed at Apo(a). For optimal selection of individuals that would benefit from specific therapy, it becomes imperative to measure the target protein too. The same principle applies to therapy monitoring. Consequently, to enable personalized CVD patient management at the molecular level, measurement of molecularly defined apolipoproteins is needed.

### Why measure apolipoproteins as a panel?

Lipids are metabolized in a complex human system with a dynamic continuum of lipoproteins. Therefore, the measurement of only a couple of biomarkers, such as LDL-c and TG, oversimplifies the individual’s lipid metabolic status and captures only a fragment of a patient’s cardiovascular risk. As previous chapters already pointed out, apolipoproteins are almost all interconnected, thereby underscoring the rationale of measuring apolipoproteins as a multiplex panel. Their functionality and effect on cardiovascular risk is dependent on what lipoprotein particles they reside on. For example, ApoC-I can reside on HDL, employing a cardioprotective role or on TGRLs employing an proatherogenic role. This underscores the significance of assessing the ratios between these apolipoproteins to gain insight into their distribution, and consequently their functionality. For instance, as to ApoC-I, its presence on HDL is associated with favorable outcomes. Thus, evaluating the ratio of ApoC-I to ApoA-I could provide valuable information regarding the distribution of ApoC-I on HDL. Similarly, the ApoC-I to ApoB ratio could offer insights into the distribution of ApoC-I on TGRLs. This dual approach, considering both ApoC-I to ApoA-I and ApoC-I to ApoB ratios, may offer a comprehensive view of the apolipoprotein functionality and distribution across lipoprotein subclasses. Another example is given by ApoE of which its phenotype exerts different properties impacting the lipid metabolism. ApoE2 for example binds with a lower affinity to the hepatic clearance receptors than the other isoforms, affecting the concentration of ApoE2-containing lipoprotein particles. The interaction between ApoE and the receptors responsible for the clearance of lipoprotein particles has an impact on lipid metabolism, and subsequent cardiovascular risk [112]. Therefore, it is important to co-determine the individual’s ApoE phenotype in the multiplex apo panel, as some phenotypes carry a higher risk than others. Given these considerations, adopting a multiplex approach for measuring apolipoproteins and for ApoE phenotyping provides an informative and diagnostic procedure that will also remain valuable to follow the results of therapy.

### Why mass spectrometry is the preferred analytical method to quantify apolipoproteins?

As mentioned in the previous section, apolipoproteins should be measured as part of a panel. This calls for a mass spectrometry (MS)-based approach, which enables multiplex testing in contrast to the more conventional immunoassay-based tests. Immunoassay-based tests quantifying apolipoproteins have been implemented in clinical practice for Apo(a), ApoB and ApoA-I. So why measure apolipoproteins with an MS test that requires a relatively complex (pre-)analytical phase? This can be explained by a couple of examples. First, MS allows the direct measurement of proteotypic peptides, in contrast to monoclonal/polyclonal immunoassays which depend on the binding specificity of antibodies towards unique, non-repetitive epitopes, resulting in an indirect measurement. Secondly, MS enables multiplex testing making this the preferred approach when measuring a panel of proteins that are interrelated as a part of one complex biological system, such as lipid metabolism. In addition, MS offers a certain level of flexibility, as extending an existing protein panel with a newly identified clinically relevant protein is relatively easy as compared to developing a new immunoassay test. Thirdly, quantification of Lp(a), through measurement of Apo(a), by immunoassays has proven to be flawed due to the heterogeneity of Apo(a) isoforms in both patient specimens and calibrators [8, 175–177]. Immunoassay tests often use latex-bound polyclonal antibodies that are reported to detect the repeating KIV2 of Apo(a), making their results Apo(a) isoform dependent, resulting in large between-method variation [178]. The MS-based test is an Apo(a) isoform independent test by design as the selected Apo(a) quantifying proteotypic peptides are KIV2 independent, providing high analytical specificity, thus eliminating the difficulties associated with varying Apo(a) isoforms [179]. Finally, besides protein quantification, MS enables qualitative assessment of proteins through the identification of isoforms, mutations, glycosylations and other post-translational modifications, as well [178, 180, 181]. In summary, MS is not affected by the challenges that immunoassay-based tests are facing [179].

In light of these considerations, the preference for an MS-based approach for an apo-panel assay becomes clear, despite the complexity associated with its initial (pre)-analytical phases. We and others have developed MS-based tests to accurately quantify serum apolipoproteins [182–187]. Our lab-developed apo-panel test enables multiplex quantification of nine apolipoproteins, including Apo(a), ApoB-100, ApoC-I, ApoC-II, ApoC-III, ApoE, ApoA-I, ApoA-II, and ApoA-IV with stable performance documented for up to four years at least [188, 189]. Additionally, the test allows ApoE phenotyping (ApoE2, ApoE3 and ApoE4 phenotypes), with a performance identical to ApoE genotyping [181].

## Conclusion: the path to adoption and implementation of apolipoproteins for personalized CVD patient management

**Fig. 1:**
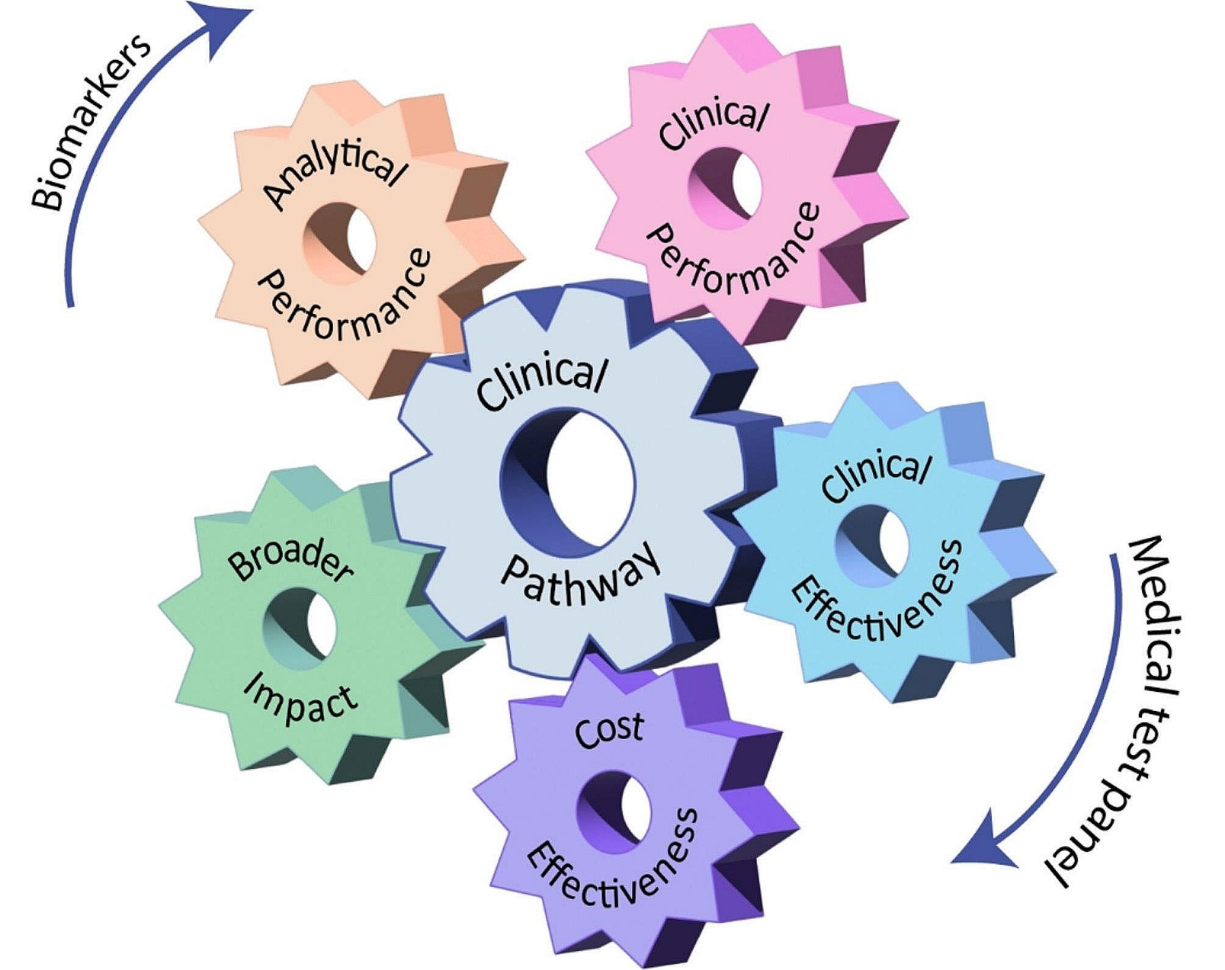
Test evaluation framework for multiplex apolipoprotein testing in cardiovascular patient management. Framework adapted from Horvath et al. [190].

To implement a new medical test in the clinic, all five key elements of the cyclic test evaluation framework constructed by the EFLM Test Evaluation Working Group [190], must be evaluated (Fig. 1).

All five aspects of test evaluation are interconnected, centered around the clinical care pathway. This framework departs from the identified unmet clinical needs, which in this case is the extensive residual cardiovascular risk beyond optimal lipid lowering therapy and the overlooked, neglected and ignored interindividual variability [16]. Through improved analytical performance of apolipoproteins compared to traditional lipid measurements, better risk prediction, diagnosis, and accurate monitoring of treatment effects of therapies directly targeting the affected molecule, we anticipate to achieve improvements of cardiovascular patient management [44]. The current lipid panel alone does not give sufficient clues for refined diagnosis and tailored therapy in case of dyslipidemia. A more personalized approach is needed and it is anticipated that apolipoproteins are promising candidates to fill this gap. The analytical performance of the multiplex apolipoprotein panel has been demonstrated [182] and the scientific validity and clinical relevance has been described in this review. Whether the apolipoprotein panel is clinically effective and predicts patient outcome awaits the results of the apolipoprotein panel measured in the ODYSSEY OUTCOMES trial, an RCT in patients with recent acute coronary syndrome [9]. In line with the earlier quote from Kohli-Lynch in collaboration with Sniderman [46]: The question is no longer what apolipoproteins add to the lipid panel, but whether the lipid panel adds anything to apolipoproteins.

**Fig. 2:**
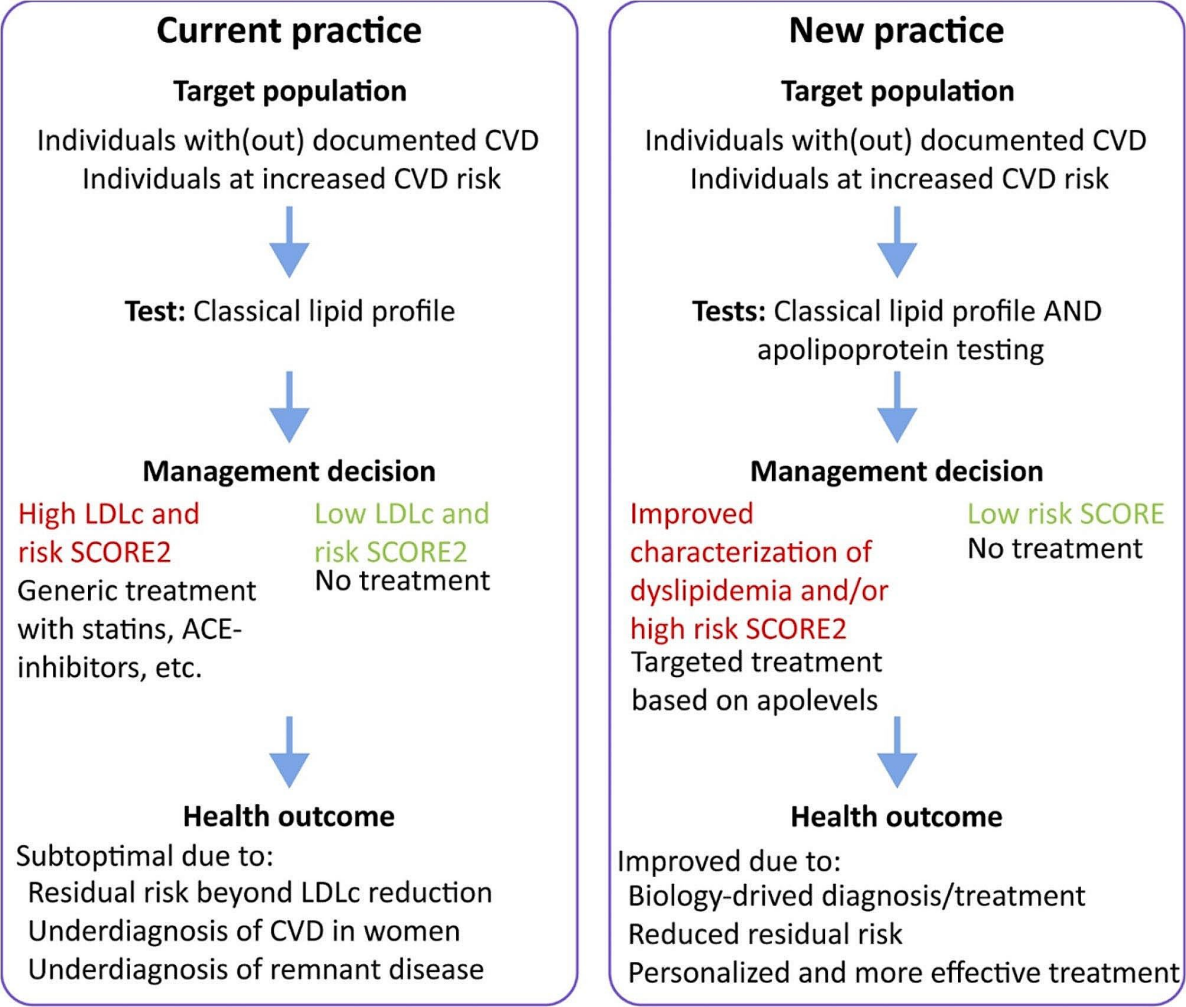
Clinical test-treatment pathways for CVD reduction according to current and new practices. Reproduced from Ruhaak et al. [16].

It is now clear from the remaining residual CV risk that the clinical test-treatment pathway for cardiovascular patient management requires major improvements, as the tests and the drugs work on average but do not work effectively in all individual patients. Definition of the cardiovascular risk at the molecular level, through the aid of apolipoproteins, in combination with therapy targeting the specific molecular defects, will improve patient outcome and enable the introduction of precision medicine for cardiovascular patient management (Fig. 2).

## Data Availability

Not applicable.

## Abbreviations

ABCA1: ATP-binding cassette transporter A1
ACS: Acute coronary syndrome
Apo: Apolipoprotein
ASCVD: Atherosclerotic cardiovascular disease
CAD: Coronary artery disease
CETP: Cholesteryl ester transfer protein
CHD: Coronary heart disease
CM: Chylomicron
CVD: Cardiovascular disease
EMA: European Medicine Agency
ER: Endoplasmic reticulum
FCS: Familial chylomicronemia syndrome
FDA: Food and Drug Administration
FFA: Free fatty acids
FH: Familial hypercholesterolemia
HDL: High-density lipoprotein
HDL-c: High-density lipoprotein cholesterol
HL: Hepatic lipase
HSPG: Heparan sulfate proteoglycan
HTG: Hypertriglyceridemia
IDL: Intermediate density lipoprotein
IMT: Intima media thickness
LCAT: Lecithin–cholesterol acyltransferase
LDL: Low-density lipoprotein
LDL-c: Low-density lipoprotein cholesterol
LDLR: Low-density lipoprotein receptor
LOF: Loss of function
Lp(a): Lipoprotein(a)
LPL: Lipoprotein lipase
LRP1: Low-density lipoprotein-like receptor protein-1
MACE: Major adverse cardiovascular events
MS: Mass spectrometry
MTP: Microsomal triglyceride transfer protein
PCSK9: Proprotein convertase subtilisin/kexin type
RCT: Randomized controlled trials
SR-B1: Scavenger receptor class B type 1
T2DM: Type 2 diabetes mellitus
TC: Total cholesterol
TG: Triglycerides
TGRL: Triglyceride-rich lipoproteins
VDLR: Very-low-density lipoprotein receptor
VLDL: Very-low-density lipoprotein
